# Extraction socket sealing using palatal gingival grafts and resorbable collagen membranes

**DOI:** 10.1186/s40902-017-0137-x

**Published:** 2017-12-25

**Authors:** Sang-Yun Kim, Young-Kyun Kim, Hyun-Suk Kim, Pil-Young Yun, Su-Gwan Kim, Yong-Hun Choi

**Affiliations:** 1 Department of Oral and Maxillofacial Surgery, Section of Dentistry, Seoul National University Bundang Hospital, 300 Gumi-dong, Bundang-gu, Seongnam, Gyeonggi-do South Korea; 2 Department of Dentistry & Dental Research Institute, School of Dentistry, Seoul National University, Seoul, South Korea; 3 Department of Oral and Maxillofacial Surgery, School of Dentistry, Chosun University, Gwangju, South Korea; 4 Department of Conservative Dentistry, Seoul National University Bundang Hospital, Seongnam, South Korea

**Keywords:** Membrane, Palatal gingival graft, Socket sealing

## Abstract

**Background:**

Socket sealing surgery is performed for the preservation of the form and volume of the soft tissue by covering the resulting socket with autogenous soft tissue graft or membrane barriers. This procedure is usually necessary to improve the esthetic results of the maxillary anterior or premolar areas.

**Methods:**

This study retrospectively investigated cases involving the open membrane technique or socket sealing surgery with a palatal gingival graft or collagen membrane where implant placement and bone grafting were performed immediately after tooth extraction. From January 2005 to December 2008, socket sealing surgery was performed in 24 patients, and 25 implants were placed.

**Results:**

All implants were successful in the follow-up period. In the palatal gingival graft group, the mean marginal bone loss was 1.17 mm during the mean follow-up period of 81.0 months. In the collagen membrane group, the mean marginal bone loss was 1.23 mm during the mean follow-up period of 76.9 months. There was no significant difference between the two groups.

**Conclusions:**

Consequently, socket sealing surgery is effective at minimizing the loss of soft tissue and alveolar bone.

**Electronic supplementary material:**

The online version of this article (10.1186/s40902-017-0137-x) contains supplementary material, which is available to authorized users.

## Background

After tooth extraction, alveolar bone is destroyed and an atrophic alveolar ridge is formed [1, 2]. So, the preservation of hard and soft tissues is very important to allow for restoration with prosthetics and implants. Mucoperiosteal flap elevation is generally required to facilitate filling with the bone graft and other materials, which is required to perform the primary suture for extraction socket preservation. Flap elevation induces bone resorption and causes other problems that are attributable to soft tissue retraction and cicatrization, both of which are esthetic problems [3]. Furthermore, the flap that is formed to cover the extraction socket, either partially or completely, may cause a retraction of the gingival margin of adjacent teeth, a loss of the interdental papilla, and the destruction of the keratinized gingiva [4]. Therefore, there have been reports on ridge preservation or socket sealing surgeries, in which various graft materials have been used to prevent the destruction of the tissues surrounding the extraction socket [5–7]. Among these surgeries, the socket sealing surgery preserves the shape and mass of the surrounding soft tissue. This is accomplished by placing an implant and performing the bone graft without dissecting the flap during the tooth extraction but covering the upper part with an autogenous soft tissue graft or a membrane. This surgery is mainly applied in cases of esthetic concern, such as the maxillary anterior or the premolar regions.

A previous study has shown that natural soft tissue healing at 6 weeks after tooth extraction was superior when socket sealing surgery was performed in the extraction socket along with the bone graft [8]. If the upper part of extraction socket was covered by soft tissue or membrane, there was a risk of complications such as necrosis or wound dehiscence. Therefore, vascularization is very important [9]. Another study reports that the use of a membrane to cover the extraction socket can have negative effects due to wound dehiscence, membrane exposure, and premature shedding [10, 11]. According to a recent study, however, intentional exposure of the resorbable membrane did not have a negative effect on guided bone regeneration (GBR) [12].

Other studies showed that extraction technique itself induces alveolar bone resorption regardless of whether the socket is treated with free gingival graft or bone graft [13]. There was also a study that the ridge preservation technique using xenograft in combination with collagen membrane significantly reduced the alveolar bone resorption after tooth extraction compared to extraction alone [14].

The authors conducted this study to examine the clinical prognosis and treatment results of cases where socket sealing was performed using the open membrane technique or a palatal gingival graft technique to prevent buccolingual soft tissue recession and to perform GBR. In these strategies, the implant is placed immediately after tooth extraction to allow the bone graft in the surrounding bony defect.

## Methods

### Patients

The authors conducted a retrospective study of socket sealing surgery cases in patients who received implant treatment in the Seoul National University Bundang Hospital between January 2006 and December 2008. This study was conducted under IRB approval (B-1206-160-111) granted by the Seoul National University Bundang Hospital. Socket sealing surgery was performed in 24 patients, with a total of 25 implants placed. Palatal tissue grafting was performed in 11 implants (anterior teeth 7, posterior teeth 4) of 11 patients (males 5, females 6). Nine implants were placed immediately after extraction, 2 implants were placed secondarily. For the palatal tissue grafting, a free graft was used in 10 of the cases and a pedicled graft was used for 1 case. A resorbable collagen membrane was used in 14 implants (anterior teeth 7, posterior teeth 7) of 13 patients (males 2, females 11). Ten implants were placed immediately after extraction, and 4 implants were placed secondarily. Seven Ossix® (OraPharma, Inc., PA, USA), 5 BioArm (ACE Surgical Supply Company Inc., Brockton, MA, USA), and 2 Bio-Gide (Geistlich Biomaterials, Inc., 6110 Wolhusen, Switzerland) resorbable collagen membranes were used.

### Measurement of marginal bone loss

Distances between implant shoulder and the first visible bone-implant contact (mm) were measured using PACS software (INFINITT PACS 3.0.9.1, Seoul, Korea). The clinician scored two marks designating where the crestal bone intersected the implant body as shown on the software. Mesial and distal bone losses of the implant were measured to calculate the mean marginal bone loss. Change in crestal bone height of each implant was calculated from the differences between the initial and final measurements from standardized periapical radiographs. The magnification rate was taken into consideration to compensate proportional differences between the real implant length and the length shown on the radiographs.

### Complications, success, and survival rate

The complications occurred during the follow-up period after implant placement were investigated. The Albrektsson (1998) definitions of success criteria for implants were used: (1) no persistent pain, discomfort, or paresthesia; (2) no abscess around the implant; (3) no mobility; (4) no radiolucency around the implant; and (5) less than 1 mm of annual marginal bone loss after prosthetic loading.

The survival rate was defined as the percentage of implants that remained until the final examination [15].

### Statistics

To statistically analyze the amount of marginal bone loss and complication rate between the two groups, independent sample *t* test was used (SPSS Inc., Chicago, IL, USA).

## Results and discussion

### Patients

Twenty-five implants were placed in a total of 24 patients, and the mean follow-up period was 78.7 months. Complications occurred in 8 implants (dehiscence *n* = 7, peri-implantitis *n* = 1), all minor and treatable. Overall success and survival rates were 100%. The mean marginal bone loss was 1.21 ± 0.13 mm at the final visit.

In palatal graft group, the patients’ age ranged from 23 to 62 years with a mean age of 46.7 years. Follow-up period ranged from 16.7 to 123.5 months with a mean of 81.0 months. In the resorbable membrane group, the patients’ age ranged from 23 to 76 years with a mean age of 50.5 years. Follow-up period ranged from 14.4 to 122.1 months with a mean of 76.9 months. When palatal tissue grafts were used, the marginal bone loss was 1.17 ± 0.13 mm. When a resorbable membrane was used, the marginal bone loss was 1.23 ± 0.14 mm. Significant difference was not found between the two groups (*p* > .05).

Both groups showed 100% success and survival rates (Tables 1 and 2). In the palatal graft group, there were two cases of wound dehiscence and one case of peri-implantitis, resulting in a total of 27.3% of complication rate. In resorbable membrane group, there were five cases of wound dehiscence (35.7%). There was no statistically significant difference in the incidence of complications between the two groups (*p* > .05).

**Table 1:** Socket sealing with palatal tissue grafting

Case	A	S	Area	Surg.	Implant	D	L	Bone graft	Types of palatal tissue	Comp	Healing	F/U	BL
1	52	F	#24	Ext. IP	Implantium	3.8	14	Biocera	Free	No	5.5	114.2	1.15
2	61	F	#22	Ext. IP	GS II	3.5	15	Bio-Oss	Free	No	5.6	16.7	1.2
3	23	M	#25	Ext. IP	GS II	4	15	Bio-Oss	Free	WD	6.4	45.3	1
4	49	F	#15	Ext. IP	Implantium	3.8	14	Biocera	Free	WD	3.5	99.2	1
5	40	F	#12	Ext.DP	Implantium	3.4	14	Biocera	Pedicled	No	4.3	123.5	1.1
6	47	F	#22	Ext. IP	TiUnite	3.3	15	Bio-Oss	CT	No	5.0	120.6	1.3
7	34	M	#11	Ext. IP	GS II	5	15	OrthoII Bio-Oss	Free	No	5.5	78.0	1
8	58	M	#25	Ext. IP	GS II	4	13	Biocera	Free	No	4.2	37.0	1.25
9	62	M	#22	Ext. IP	US II	3.75	15	BBP	Free	No	5.3	86.3	1.3
10	27	M	#22	Ext. IP	Implantium	3.8	14	Bio-Oss	Free	PI	5.1	105.2	1.35
11	61	F	#23	Ext. IP	GS III	4.5	15	Biocera	Free	No	1.2	65.4	1.25

*A* age, *S* sex, *D* diameter, *L* length, *Comp.* complication, *WD* wound dehiscence, *PI* peri-implantitis, *healing* healing period between the first implant surgery and the prosthetic treatment (months), *F/U* follow-up (months), *BL* bone loss (mm), *surg.* type of surgery (*Ext.* extraction, *IP* immediate implant placement, *DP* delayed placement), *GS II* (OSSTEM IMPLANT Co., Busan, Korea), *GS III* (OSSTEM IMPLANT Co., Busan, Korea), *US II* (OSSTEM IMPLANT Co., Busan, Korea), *Implantium* (Dentium, Seoul, Korea), *TiUnite* (Nobel Biocare, Gthenburg, Sweden)

**Table 2:** Socket sealing using resorbable collagen membranes

Case	A	S	Area	Surg.	Implant	D	L	Bone graft	Types of membrane	Comp	Healing	F/U	BL
1	57	F	#35	Ext. IP	Implantium	3.8	14	Bio-Oss	Ossix	No	2.5	122.1	1.25
2	23	F	#24	Ext. IP	GS II	3.5	15	AutoBT	Ossix	No	4.9	45.2	1.45
3	72	M	#11	Ext. IP	Implantium	4.3	12	Bio-Oss	Ossix	WD	7.7	100	1.1
4	25	F	#22	Ext. IP	GS II	3.5	15	Bio-Oss	Ossix	No	5.2	55.3	1.1
5	50	F	#26 #27	Ext. DP	Oneplant Oneplant	4.3 4.3	13 11.5	Bio-Oss Ortho II	Ossix Ossix	WD WD	5.6 5.6	112.9 112.9	1.3 1.1
6	36	F	#11	Ext. DP	3I	4	13	Bio-Oss	Bio-Gide	No	6.7	65.1	1.2
7	42	F	#14	Ext. DP	Oneplant	4.3	13	BBP	Bioarm	No	4.4	76.0	1.35
8	50	M	#22	Ext. IP	GS III	4	13	Biocera	Bioarm	WD	18	75.4	1.4
9	52	F	#17	Ext. IP	GS II	5	13	Biocera	Bioarm	No	3.4	96.1	1.25
10	47	F	#21	Ext. IP	Implantium	4.3	14	Biocera	Bioarm	No	3.4	73.6	1.3
11	57	F	#15	Ext. IP	CMI	4	13	Biocera	Ossix	No	4.2	72.2	1
12	76	F	#13	Ext. IP	GS III	4	13	AlloBT	Bio-Gide	WD	4.7	56.0	1.1
13	69	F	#12	Ext IP	GS II	4	15	Biocera	Bioarm	No	4.0	14.4	1.4

*A* age, *S* sex, *D* diameter, *L* length, *Comp.* complication, *WD* wound dehiscence, *PI* peri-implantitis, *healing* healing period between the first implant surgery and the prosthetic treatment (months), *F/U* follow-up (months), *BL* bone loss (mm), *surg*: type of surgery (*Ext.* extraction, *IP* immediate implant placement, *RP* ridge preservation, *DP* delayed placement), *GS II* (OSSTEM IMPLANT Co., Busan, Korea), *GS III* (OSSTEM IMPLANT Co., Busan, Korea), *US II* (OSSTEM IMPLANT Co., Busan, Korea), *Implantium* (Dentium, Seoul, Korea), *TiUnite* (Nobel Biocare, Gthenburg, Sweden)

### Case reports

#### Case 1: socket sealing surgery using a free palatal gingival graft (Fig. 1) (Table 1, case 6)

A 47-year-old female patient visited the hospital complaining of pain in her left maxillary lateral incisor. Mild tooth mobility and a cervical fracture were found, and a radiolucent periapical lesion was observed in panoramic radiograph. As a result, a plan was established to place an implant immediately following tooth extraction. On September 29, 2005, a flapless extraction was performed and the extraction socket was probed and 5 mm labial bony dehiscence was observed. Drilling was performed on the palatal side before the implant (Nobelbiocare TiUnite, 3.3 mm in diameter, 15 mm in length) was placed and connected with a cover screw. Osstell Mentor (Integration Diagnostics AB, Göteberg, Sweden) was used to measure its primary stability, which had implant stability quotient (ISQ) value of 72. A periosteal elevator was used to form a pouch in the upper part of the labial cortical bone before the Bio-Oss (Geistlich Pharma AG, Wolhausen, Switzerland) was grafted. The free palatal gingiva was then taken before the upper part of the implant was covered and sutured to install a temporary flipper. Post-surgical wound healing after the surgery was favorable, and the implant was exposed in the second surgery on February 27, 2006. Using the Osstell Mentor for measurement, the implant’s secondary stability (ISQ) was 73. On April 11, 2006, the final prosthesis was installed and it remained stable even after 121 months of function.

**Fig. 1:**
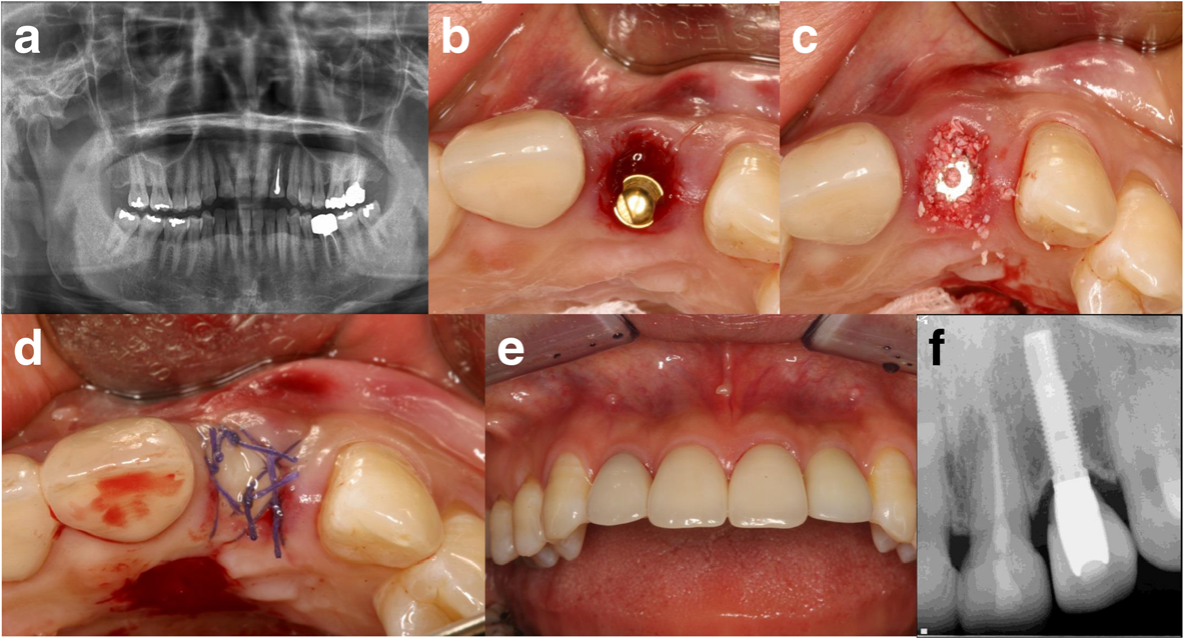
Socket sealing surgery using a free palatal gingival graft. **a** The first panoramic radiograph. **b** Implant placement. **c** Bone graft. **d** Palatal free gingival graft. **e** Intraoral view 60 months after final prosthesis placement. **f** Periapical radiograph 84 months after final prosthesis placement

#### Case 2: socket sealing using a pedicled palatal gingival graft (Fig. 2) (Table 1, case 5)

A 40-year-old female patient visited the hospital with mobility and pain in right maxillary lateral incisor. Clinical and radiological examinations revealed severe destruction of the surrounding alveolar bone. On July 28, 2005, extraction and socket curettage were performed before Biocera (Osscotec, Seoul, Korea) was transplanted. A pedicled flap was formed on palatal side to maintain labial soft tissue contours. The flap was then coronally positioned towards the extraction socket prior to suturing. Five months later, a palatal crestal incision and a flap elevation were performed before implant placement. A bone graft (Orthoblast II: Orthobiologics, Irvine, USA) was placed on the labial side of the implant, a collagen membrane (Ossix: Orapharma Inc., Louis Drive Warminster, PA, USA) was covered, and the wound was sutured. On May 9, 2006, a second surgery was performed. The final prosthesis was installed on June 27, 2006. This prosthesis remained stable even after 124 months of function.

**Fig. 2:**
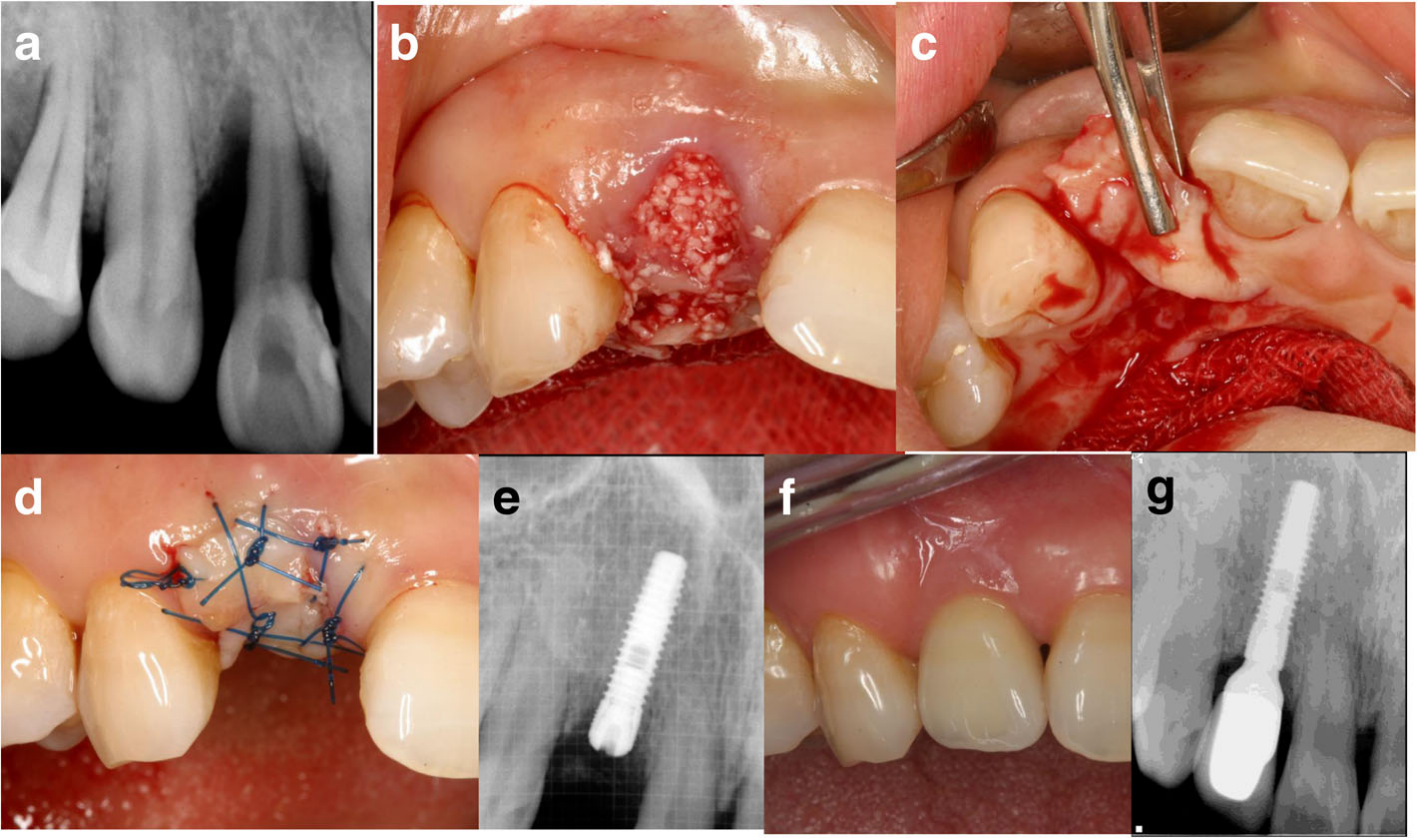
Socket sealing using a pedicled palatal gingival graft. **a** The first periapical view. **b** Extraction and bone graft. **c**, **d** Pedicled palatal flap. **e** Periapical radiograph after implant placement. **f** Intraoral view 58 months after final prosthesis placement. **g** Periapical radiograph 93 months after final prosthesis placement

#### Case 3: socket sealing with a resorbable collagen membrane (Fig. 3) (Table 2, case 3)

A 72-year-old male patient visited the hospital due to a right maxillary central incisor fracture. A periapical lesion was not found, and his periodontal condition was favorable. On March 6, 2007, a flapless atraumatic extraction was performed and the socket was probed. According to the probing results, the existing labial bone destruction was severe. Mucoperiosteal flap elevation was performed before implant placement. Bio-Oss was grafted to restore the labial dehiscence defect. A collagen membrane (Ossix) was then covered, and the wound was sutured. Flap undermining was not performed to maintain the labial soft tissue contour. The membrane where the upper part of the implant was covered was intentionally left exposed. The exposed membrane was absorbed over time, leading to favorable secondary healing. After 6 months, the secondary surgery was performed. The final prosthesis was installed on November 29, 2007. This prosthesis remained stable even after 100 months of function.

**Fig. 3:**
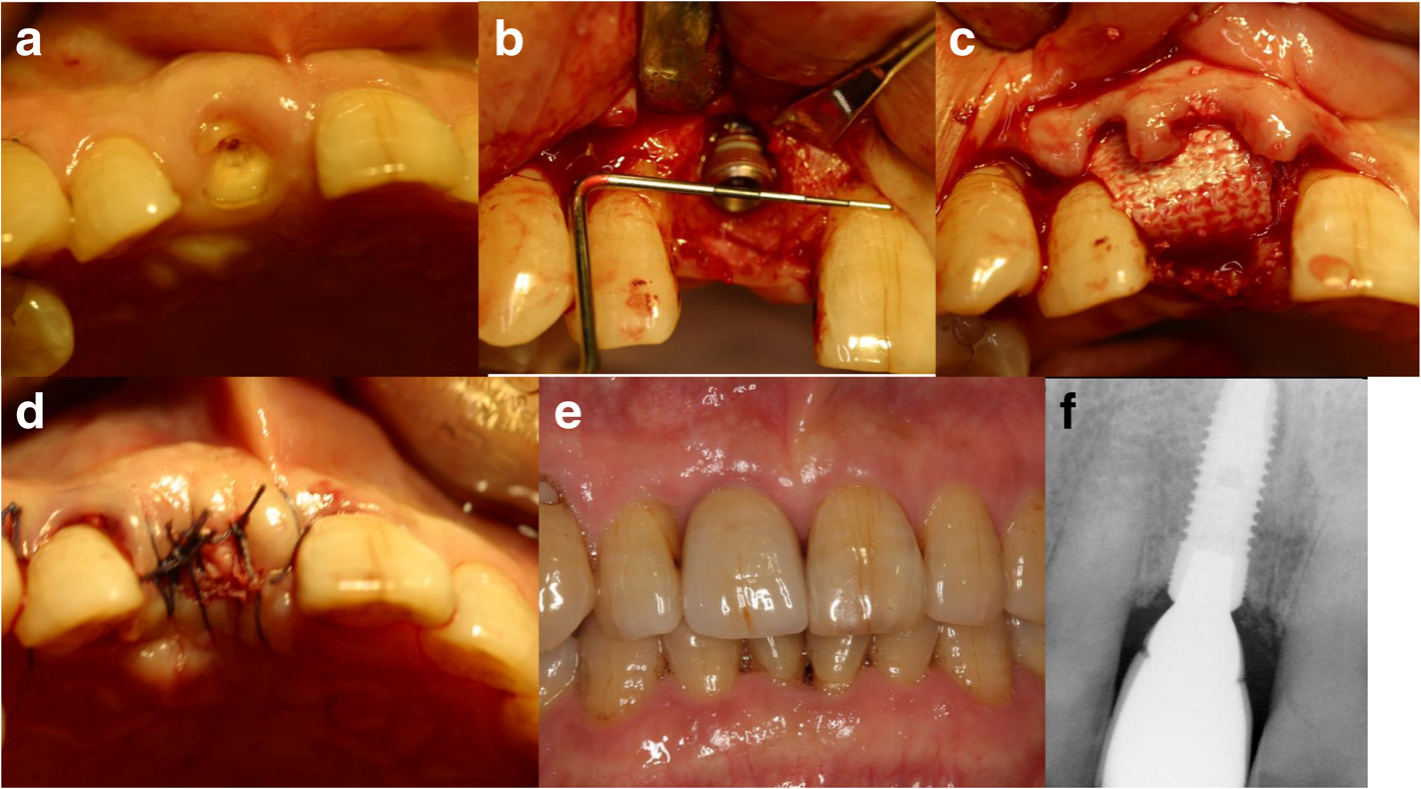
Socket sealing with a resorbable collagen membrane. **a** The first visit. **b** Implant placement. **c** Bio-Oss was transplanted to the labial dehiscence defect before the collagen membrane (Ossix) was covered and the wound was sutured. **d** Flap undermining was not performed to promote labial soft tissue contour maintenance, and the membrane where the upper part of implant was covered was intentionally left exposed. **e** Intraoral view 48 months after final prosthesis placement. **f** Periapical radiograph 72 months after final prosthesis placement

Socket sealing surgery is performed for extraction socket preservation when an implant is placed immediately following tooth extraction. Because a mucoperiosteal flap is not formed, this surgery is favorable because the alveolar bone and its surrounding soft tissues can be preserved as much as possible. In this study, an open membrane technique and a palatal gingival graft technique were used, with the aim of preserving as much keratinized gingival tissue as possible. However, the benefits of keratinized gingival preservation remain controversial. Some studies have stated that there is insufficient evidence for the importance of keratinized gingival preservation [16]. However, other studies have indicated that preservation of the vestibule and its associated keratinized gingiva can improve oral hygiene and minimize the risk of bleeding on probing, recession, plaque-induced peri-implantitis in implant, and restoration in the future [17–19]. As an attempt to cover the grafted extraction socket and to prevent bacterial colonization from salivary contamination, free gingival grafts were introduced in 1994 for socket sealing [20] However, the reported failure rate was 50% or higher (26% for total necrosis and 31% for partial necrosis) [9]. For this reason, a new procedure using a combined epithelialized-subepithelial graft was introduced [21]. The free gingival graft used in this study demonstrated that wound dehiscence occurred in two cases, but that healing was successful in all cases. All assessed grafts functioned successfully until the final prosthesis was loaded.

Wilson et al. presented clinical results for cases where a connective tissue membrane was used for immediate implant placement. They reported excellent osseointegration results with immediate implant placement in horizontal bone defects of 4 mm or higher [22]. Recently, Stimmelmayr et al. reported bone grafting to rebuild buccal alveolar defects at the same time that the tooth is extracted, combined with a soft tissue graft to seal the socket, showed promising results and could be an alternative treatment to delayed hard tissue grafting [23].

Zubillanga et al. attempted to use primary sutures for ridge preservation in all cases. They found that though 45% of the membrane was eventually exposed, infections or other clinical complications were not observed in any cases [24]. Furthermore, Engler-Hamm et al. compared the use of primary sutures made of resorbable membranes with intentional exposure during ridge preservation. They reported that the discomfort and swelling following surgery was less severe when the membrane was intentionally exposed without flap dissection than when the primary suture was performed through flap dissection. They also found that the results were more favorable when keratinized gingival tissues were preserved, and no differences in bone resorption were observed [25]. In this study, the resorbable collagen membrane was intentionally exposed to allow for socket sealing. All of the cases assessed here had successful results with minimal observed complications. Unlike previous studies that showed the resorbable membrane as a reservoir of bacterial propagation resulting in infection and eventually failure of bone graft, there was not a single case of failure due to infection when membranes were intentionally exposed. This means that appropriate antibiotic therapy and disinfection could sufficiently reduce the risk of infection, and extraction socket sealing surgery using resorbable collagen membrane can show clinical results similar to those of palatal gingival graft.

In this study, socket sealing surgery was performed to prevent soft tissue retraction in regions of esthetic importance, such as the maxillary anterior region or the premolar region. In the average observation period of 78.7 ± 31.4 months, the post-extraction loss of marginal bone was found to be 1.21 ± 0.13 mm on average, indicating that a stable state was maintained. In conclusion, palatal gingival grafts and open membrane techniques using resorbable membranes can be used to produce clinically favorable results in terms of soft tissue preservation in regions of esthetic importance (Additional file 1).

## Conclusions

Consequently, socket sealing surgery is effective at minimizing the loss of soft tissue and alveolar bone.

## Additional file

Additional file 1: Case form and result of data. (XLSX 31 kb)

## Abbreviations

GBR: Guided bone regeneration
ISQ: Implant stability quotient
